# Improved Resection and Outcome of Colon-Cancer Liver Metastasis with Fluorescence-Guided Surgery Using In Situ GFP Labeling with a Telomerase-Dependent Adenovirus in an Orthotopic Mouse Model

**DOI:** 10.1371/journal.pone.0148760

**Published:** 2016-02-05

**Authors:** Shuya Yano, Kiyoto Takehara, Shinji Miwa, Hiroyuki Kishimoto, Yukihiko Hiroshima, Takashi Murakami, Yasuo Urata, Shunsuke Kagawa, Michael Bouvet, Toshiyoshi Fujiwara, Robert M. Hoffman

**Affiliations:** 1 AntiCancer, Inc., San Diego, CA, United States of America; 2 Department of Surgery, University of California San Diego, San Diego, CA, United States of America; 3 Department of Gastroenterological Surgery, Okayama University, Graduate School of Medicine, Dentistry and Pharmaceutical Sciences, Okayama, Japan; 4 Oncolys BioPharm Inc., Tokyo, Japan; National Cancer Institute, UNITED STATES

## Abstract

Fluorescence-guided surgery (FGS) of cancer is an area of intense development. In the present report, we demonstrate that the telomerase-dependent green fluorescent protein (GFP)-containing adenovirus OBP-401 could label colon-cancer liver metastasis in situ in an orthotopic mouse model enabling successful FGS. OBP-401-GFP-labeled liver metastasis resulted in complete resection with FGS, in contrast, conventional bright-light surgery (BLS) did not result in complete resection of the metastasis. OBP-401-FGS reduced the recurrence rate and prolonged over-all survival compared with BLS. In conclusion, adenovirus OBP-401 is a powerful tool to label liver metastasis in situ with GFP which enables its complete resection, not possible with conventional BLS.

## Introduction

Fluorescence-guided surgery (FGS) is an area of currently intense research [1]. Green fluorescent protein (GFP) has been previously successfully used for labeling tumors *in situ* for FGS using a telomerase-dependent adenovirus (OBP-401) that expresses the *gfp* gene only in cancer cells [1–7]. Since recurrent cancer cells stably express GFP, detection of cancer recurrence and metastasis is also possible with OBP-401 GFP labeling [7], in contrast to fluorescent-antibody or other non-genetic labeling [1].

We have previously demonstrated OBP-401-based fluorescence-guided surgery is highly effective in various types of cancers including soft tissue sarcoma [4], glioblastoma [5], pancreatic cancer [6], and lung cancer [7].

Recently, GFP-expressing liver metastasis in an orthotopic mouse models was completely resected. In contrast, conventional bright-light surgery (BLS) could not fully resect the metastasis [8]. However, this experiment used transplanted human cancer cells that were previously engineered to express GFP in vitro.

In the present report, we demonstrate OBP-401 brightly labels colon cancer liver metastasis in an orthotopic mouse model *in situ* with GFP, enabling complete resection of liver metastasis by FGS and prolonged survival compared to BLS.

## Materials and Methods

### Ethics Statement

All animal studies were conducted with an AntiCancer Institutional Animal Care and Use Committee (IACUC)-protocol specifically approved for this study and in accordance with the principals and procedures outlined in the National Institutes of Health Guide for the Care and Use of Animals under Assurance Number A3873-1. In order to minimize any suffering of the animals, anesthesia and analgesics were used for all surgical experiments. Animals were anesthetized by intramuscular injection of a 0.02 ml solution of 20 mg/kg ketamine, 15.2 mg/kg xylazine, and 0.48 mg/kg acepromazine maleate. The response of animals during surgery was monitored to ensure adequate depth of anesthesia. Ibuprofen (7.5 mg/kg orally in drinking water every 24 hours for 7 days post-surgery) was used in order to provide analgesia post-operatively in the surgically-treated animals. The animals were observed on a daily basis and humanely sacrificed by CO_2_ inhalation when they met the following humane endpoint criteria: prostration, skin lesions, significant body weight loss, difficulty breathing, epistaxis, rotational motion and body temperature drop. The use of animals was necessary to develop fluorescence-guided surgery of liver metastasis. Animals were housed with no more than 5 per cage. Animals were housed in a barrier facility on a high efficiency particulate arrestance (HEPA)-filtered rack under standard conditions of 12-hour light/dark cycles. The animals were fed an autoclaved laboratory rodent diet (S1 Checklist).

### GFP-Expressing Telomerase-Specific Adenovirus

The GFP-expressing adenovirus OBP-401 contains the promoter element of the human telomerase reverse transcriptase (*hTERT*) gene which drives the expression of E1A and E1B genes linked to an internal ribosome entry site for selective replication only in cancer cells, and the *GFP* gene which is driven by the CMV promoter [9].

### Cell Line and Cell Culture

**Fig 1:**
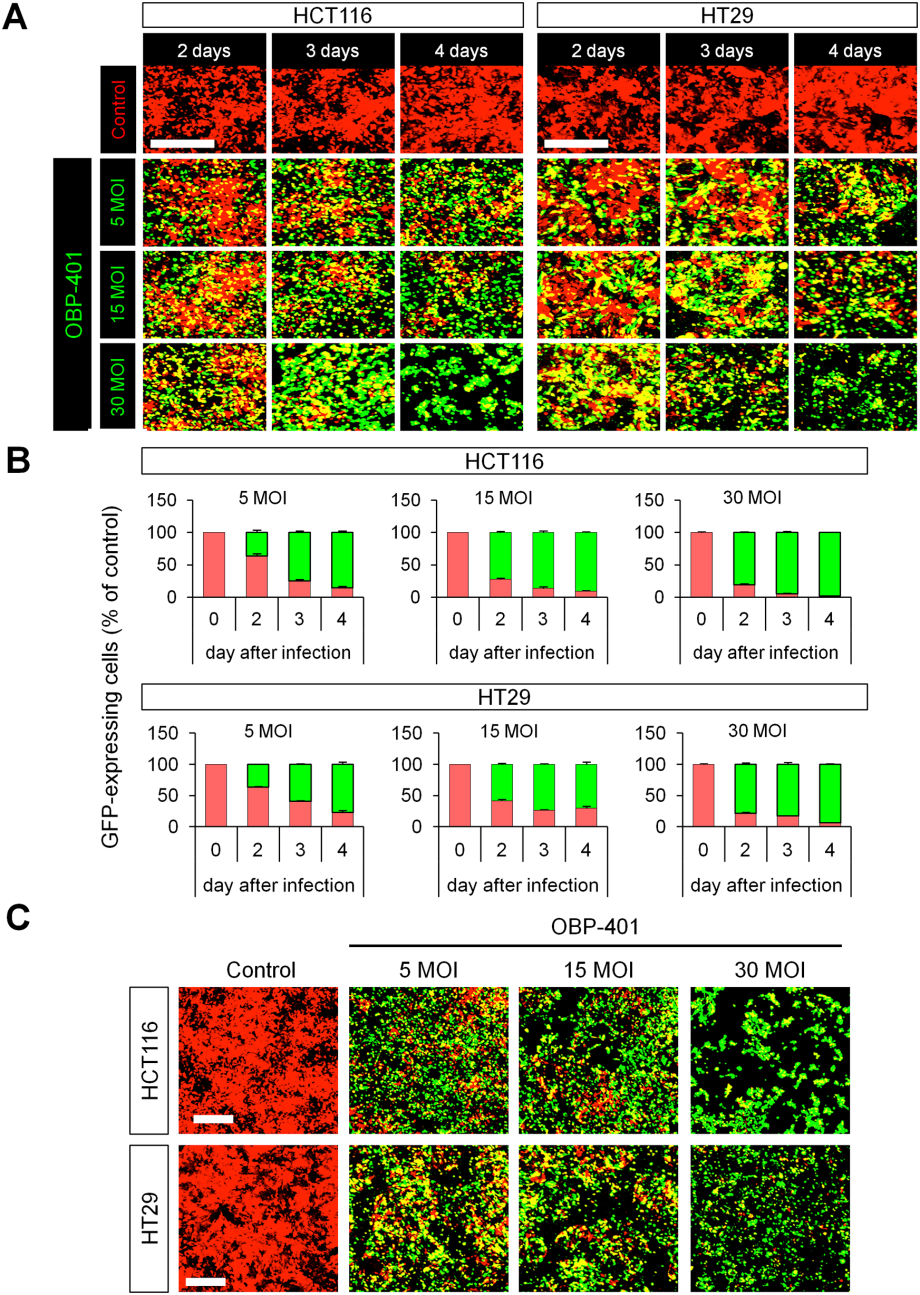
OBP-401 labels HCT-116 and HT29 colon cancer cells with GFP *in vitro*. Human colon cancer cell lines HCT-116 and HT29 expressing RFP (HCT-116-RFP and HT29-RFP, respectively) were seeded in 6-well plates (1 × 10^5^ cells / well). OBP-401 was added at the indicated multiples of infection (MOI) 24 hours after cell seeding. Images were acquired with an FV1000 confocal laser scanning microscope (Olympus, Tokyo, Japan). **A.** Representative images of non-infected HCT-116-RFP and HT29-RFP cells and HCT-116-RFP and HT29-RFP cells 2, 3, and 4 days after infection of OBP-401 at an MOI of 5, 15 and 30. **B.** Histogram shows the frequency of GFP-expressing HCT-116 and HT-29 cells at indicated days after infection with OBP-401. **C.** Representative image of HCT-116-RFP and HT29-RFP cells 5 days after infection of OBP-401 at an MOI of 5, 15, and 30.

The human colon cancer cell lines HCT-116 and HT-29 expressing RFP (HCT-116-RFP [10] and HT29-RFP [11], respectively) were maintained and cultured in DMEM with 10% fetal bovine serum (FBS) and 5% penicillin/streptomycin. OBP-401 was used to label colon cancer cells with GFP (Fig 1).

### Animal Experiments

Athymic (*nu/nu*) nude mice (AntiCancer, Inc., San Diego) were kept in a barrier facility under HEPA filtration. Mice were fed with an autoclaved laboratory rodent diet (Tecklad LM-485, Western Research Products). All animal studies were conducted in accordance with the principles and procedures outlined in the National Institutes of Health Guide for the Care and Use of Laboratory Animals under Assurance Number A3873–01.

### Liver Metastasis Model

For development of high liver-metastatic colon cancer cells, HCT-116-RFP cells (2 × 10^6^) were injected into the spleen of female arthymic nude mice (5 weeks old). HCT-116-RFP cells produced experimental liver metastasis one month after injection. The experimental liver metastasis were harvested and re-injected into the spleen. High-metastatic colon cancer cells, termed HCT-116L3-RFP, were selected after three such cycles. An orthotopic solitary-metastatic model was developed with HCT-116-L3-RFP cells implanted under the serosa in the liver of nude mice. The orthotopic metastases readily grew in the liver. Tumor growth was followed by RFP fluorescence using noninvasive fluorescence imaging.

### *In Vitro* and *In Vivo* Imaging

Time-course imaging of OBP-401 labeling of HCT-116-RFP, HCT-116L3-RFP and HT-29-RFP cells in vitro was performed with an FV1000 confocal laser-scanning microscope (Olympus, Tokyo, Japan) [12]. For whole-body or whole-tumor imaging, the OV100 small animal imaging system (Olympus) [13], was used.

### OBP-401-Based Fluorescence-Guided Surgery (FGS) (OBP-401- FGS)

All animal procedures were done under anesthesia using s.c. administration of the ketamine mixture, described above. The experimental liver metastasis, labeled with GFP by OBP-401 (1 × 10^8^ PFU), was imaged using the OV100 before surgery. FGS was performed under GFP guidance using either a hand-held DinoLite fluorescence imaging system (AM4113T-GFBW Dino-Lite Premier; AnMo Electronics Corp, Taiwan) [14,15] or the stationary Illumatool imaging system (Lightools Research, Encinitas, CA) [16] which enabled precise location of the liver metastasis and surgical-resection beyond the tumor margin. After surgery, it was determined whether there were residual cancer cells or not. If there were residual cancer cells, an additional resection was performed.

### Statistical Analysis

Data are shown as means ± SD. For comparison between two groups, significant differences were determined using Student’s t-test. Pearson chi-square analysis was used to compare the rate of recurrence between BLS and OBP-401-FGS. Statistical analysis for over-all survival was performed using the Kaplan-Meier test along with log-rank test. *P* values of < 0.05 were considered significant.

## Results and Discussion

### GFP-Expressing Adenovirus OBP-401 Labels Human Colon Cancer Cells In Vitro

Time-course imaging showed that OBP-401 labeled RFP-expressing HCT-116 (HCT-116-RFP) and HT29 (HT29-RFP) colon cancer cells with GFP (Fig 1A) in a dose-dependent manner (Fig 1B). GFP fluorescence after OBP-401 infection of HCT-116-RFP and HT29-RFP cancer cells became sufficiently bright from day 2 to day 4 such that the cells appeared green (Fig 1A). Moreover, high dose OBP-401 also killed HCT-116-RFP and HT29-RFP cancer cells (Fig 1C).

### Orthotopic liver metastasis model

**Fig 2:**
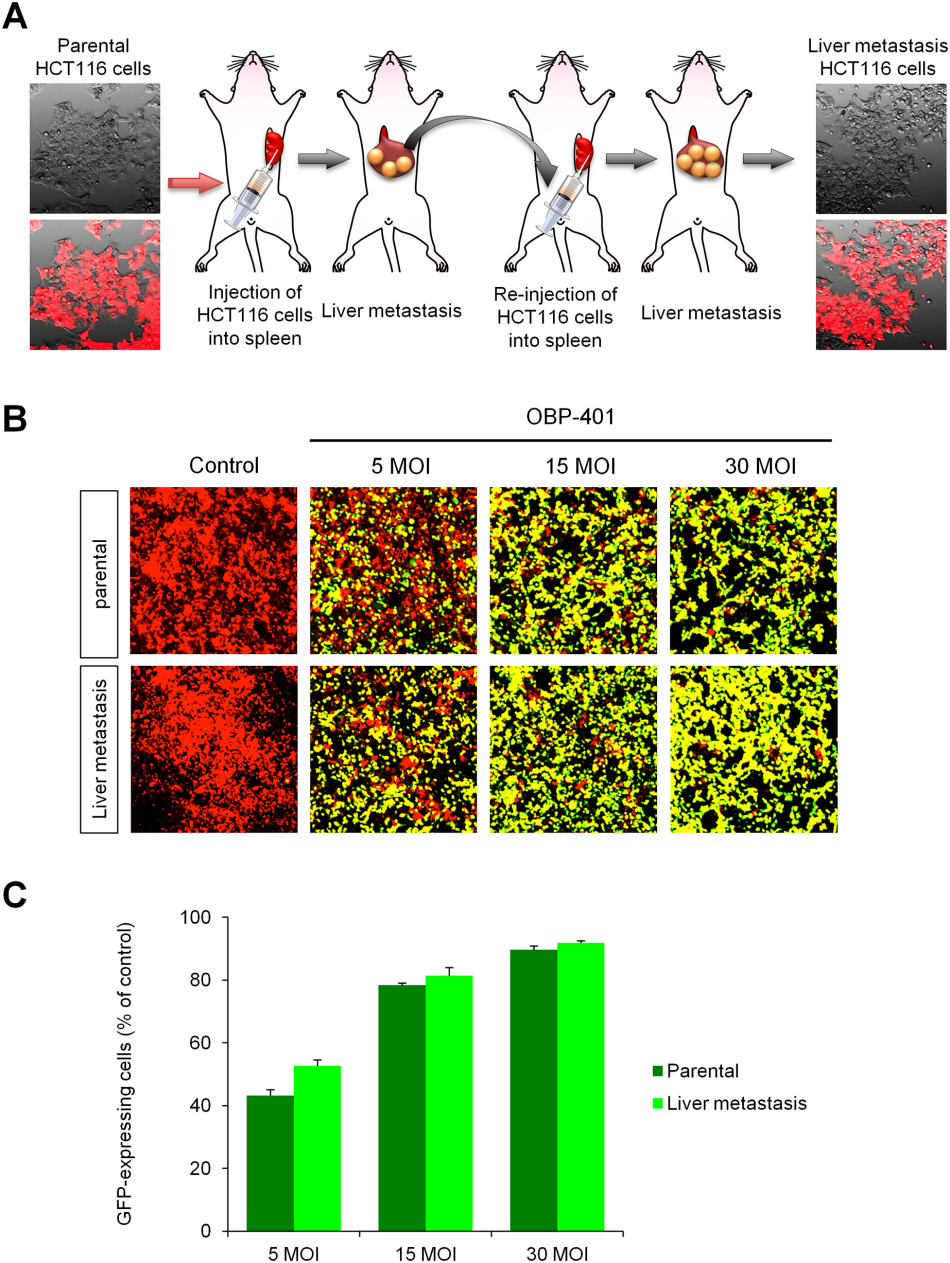
OBP-401 labels high-metastatic colon-cancer-cell selected variants. **A.** Scheme of selecting colon cancer high-liver-metastasis cells. HCT-116-RFP cells (2 × 10^6^) were injected into the spleen of female arthymic nude mice (5 weeks old). After 3 cycles of transplantation and harvest, high liver-metastatic colon cancer cells were selected and termed HCT-116L3-RFP. Images were acquired with the FV1000 confocal laser scanning microscope. **B.** HCT-116-RFP and HCT-116L3-RFP cells were seeded in 6-well plates (1 × 10^5^ cells / well). OBP-401 was added at the indicated multiples of infection (MOI) 24 hours after cell seeding. Images were acquired with the FV1000 confocal laser scanning microscope. Representative images of non-infected HCT-116-RFP and HCT-116L3-RFP cells, and HCT-116-RFP and HCT-116L3-RFP cells 3 days after infection of OBP-401 at an MOI of 5, 15 and 30. **C.** Histogram shows the frequency of GFP-expressing HCT-116-RFP and HCT-116L3-RFP cells at indicated days after infection of OBP-401. Data are shown as average ± SD. N = 5.

For development of high liver-metastatic colon cancer cells, HCT-116-RFP cells (2 × 10^6^) were injected into the spleen of female athymic nude mice (5 weeks old). HCT-116-RFP cells produced experimental liver metastasis within one month after injection. The experimental liver metastasis were harvested and re-injected into the spleen. High-metastatic colon cancer cells, termed HCT-116L3-RFP, were selected after three such cycles (Fig 2A). Time-course imaging showed that OBP-401 labeled HCT-116L3-RFP brightly with GFP in a dose dependent manner *in vitro* (Fig 2B and 2C).

**Fig 3:**
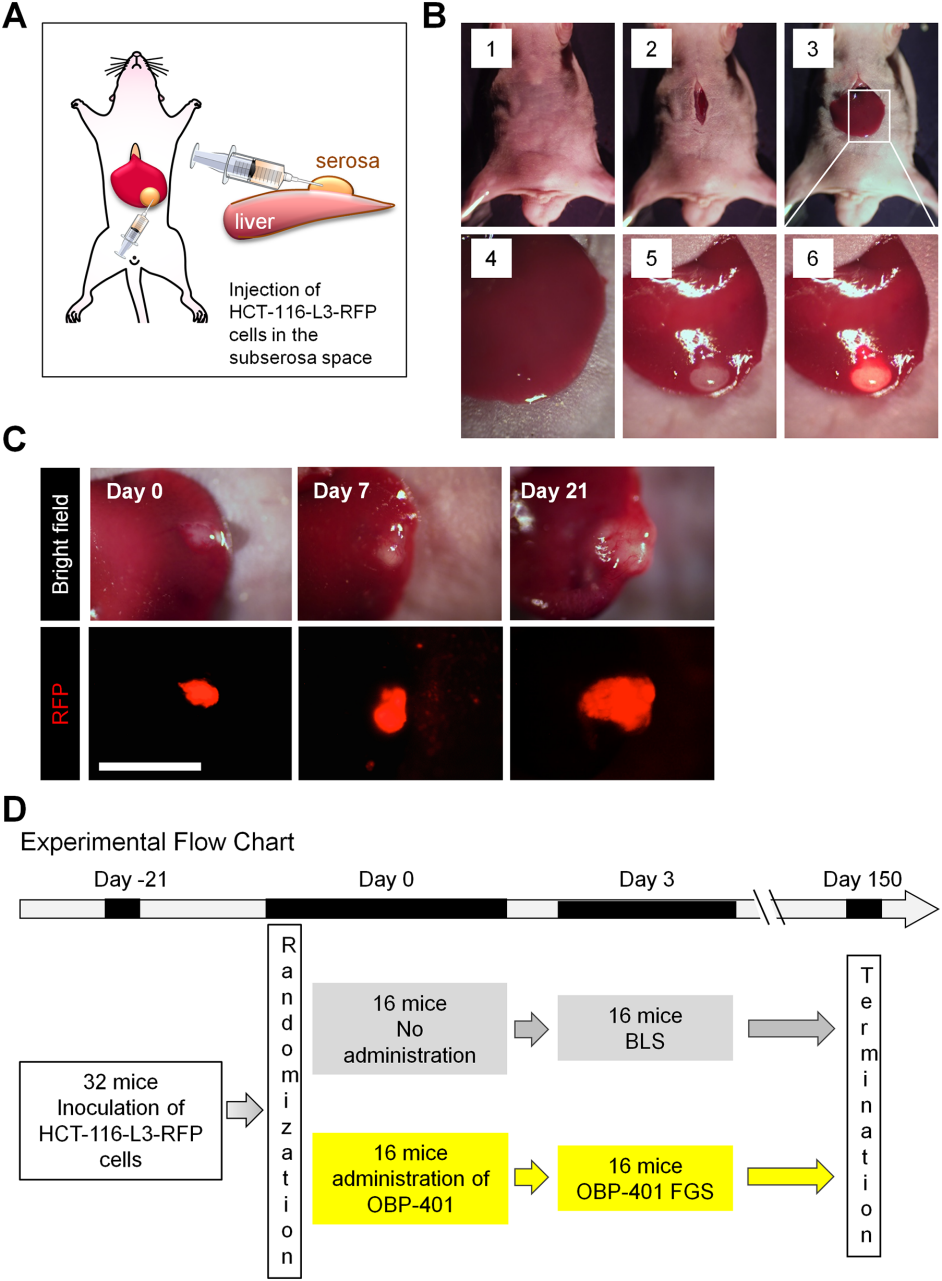
Establishment of solitary liver-metastasis model. **A.** Scheme for establishing solitary live metastasis. **B.** HCT-116L3-RFP cells (2×10^5^), in Matrigel (BD), were inoculated in the subserosa space of the liver of nude mice (5 weeks old). Panels 1, 2. Open abdominal wall. Panel 3. Exteriorizing the liver. Panel 4. Injection of HCT-116L3-RFP cells in the subserosa of the liver. Panel 5. RFP-fluorescence image of injected HCT-116L3-RFP cells in the subserosa of the liver. Panel 6. Merged image of injected HCT-116L3-RFP cells in the subserosa of the liver. **C.** Time-course imaging of liver metastasis growth. Upper panels; low magnification images. Lower panels; high magnification images. **D.** Experimental flowchart with endpoints.

An orthotopic solitary-metastatic model was developed with HCT-116-L3-RFP cells, in Matrigel, implanted under the serosa in the liver of nude mice (Fig 3A and 3B). The orthotopic metastases readily grew in the liver. Tumor growth was followed by RFP fluorescence using noninvasive fluorescence imaging (Fig 3C). After liver-metastasis growth for 21 days, the mice were randomized into two groups; BLS and OBP-401-FGS (Fig 3D).

### BLS Results in Incomplete Resection of Solitary Liver Metastasis

**Fig 4:**
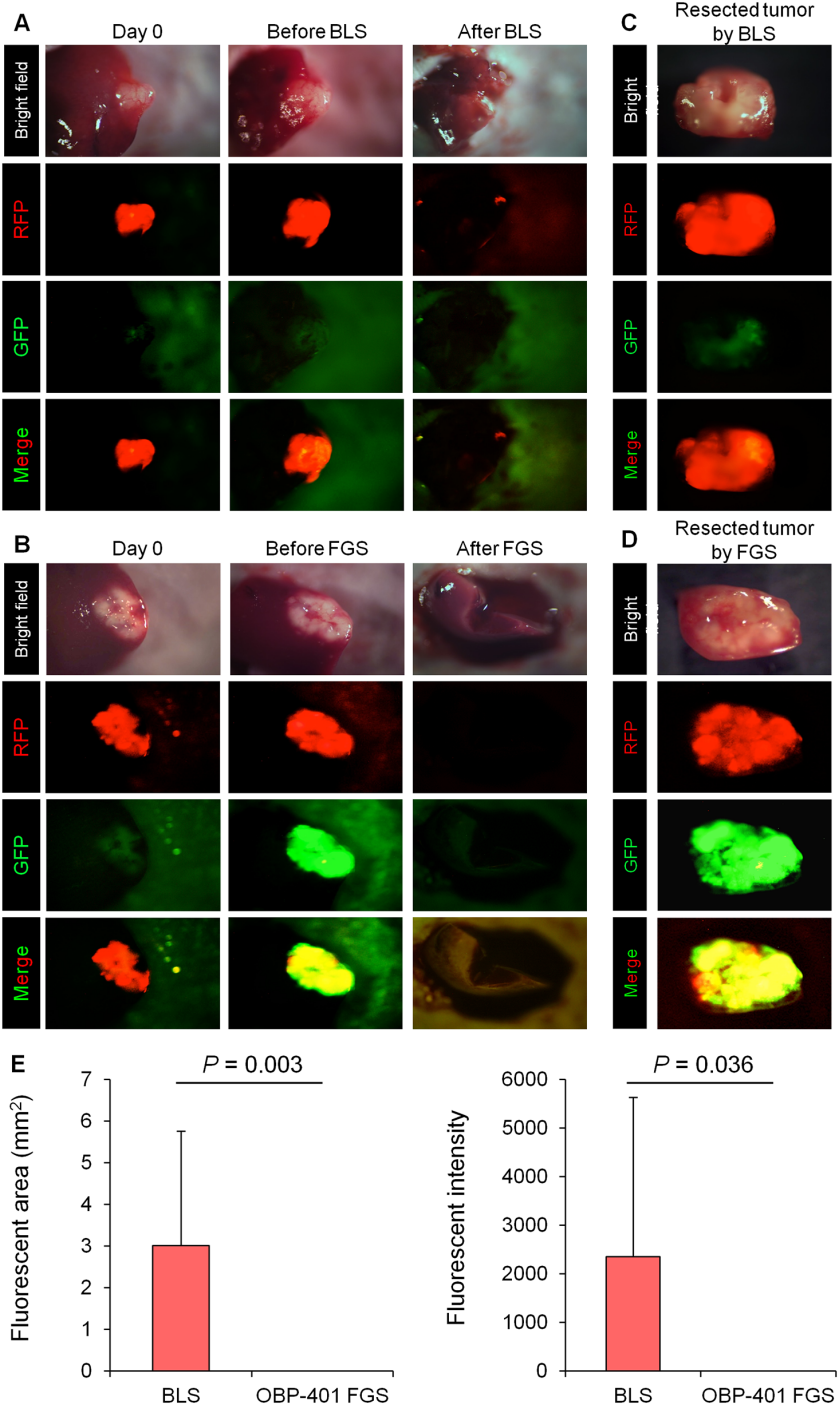
Comparison of OBP-401-based fluorescence-guided surgery with bright light surgery of a solitary liver metastasis. OBP-401 was injected intra-tumorally at 1 × 10^8^ PFU when tumors reached approximately 100 mm^3^ (6 mm diameter). **A.** Representative whole-liver image of non-infected liver metastasis before and after bright-light surgery (BLS). **B.** Representative whole-liver image of liver metastasis before injection of OBP-401 and before and after OBP-401-based FGS using the Illumatool imaging system [16]. **C.** Representative whole-tumor image of resected tumor using BLS. **D.** Representative image of entire resected tumor using OBP-401-FGS. **E.** Bar graph shows the comparison of the fluorescent area in the surgical bed after BLS or OBP-401-FGS (left). Bar graph shows the comparison of fluorescence intensity in the surgical bed after BLS or OBP-401-FGS (right). Fluorescence intensity and area are calculated with ImageJ software. Data are shown as average ± SD. N = 16.

We performed bright-light surgery (BLS) on orthotopic solitary liver metastases (Fig 4A). The tumor margin was invisible in the deep area of the liver under bright light, and thus RFP-expressing metastatic colon cancer cells remained after BLS (Fig 4A and 4D).

### OBP-401-FGS of Solitary Liver Metastasis Results in Complete Resection

Solitary metastases were resected 3 days after i.t. injection of OBP-401 (1 × 10^8^ PFU) (Fig 4B). OBP-401 conferred GFP fluorescence to solitary metastases which was sufficiently bright to perform complete resection using the stationary Illumatool in vivo fluorescence imaging system (Fig 4B and 4D). Tumor imaging showed that OBP-401 GFP and the RFP fluorescence of the metastasis co-localized (Fig 4B). OBP-401-GFP-based FGS resulted in no detectable residual metastatic cancer cells (Fig 4B and 4E).

### OBP-401-FGS of Solitary Liver Metastasis Using a Hand-Held Portable Fluorescence Imaging System

**Fig 5:**
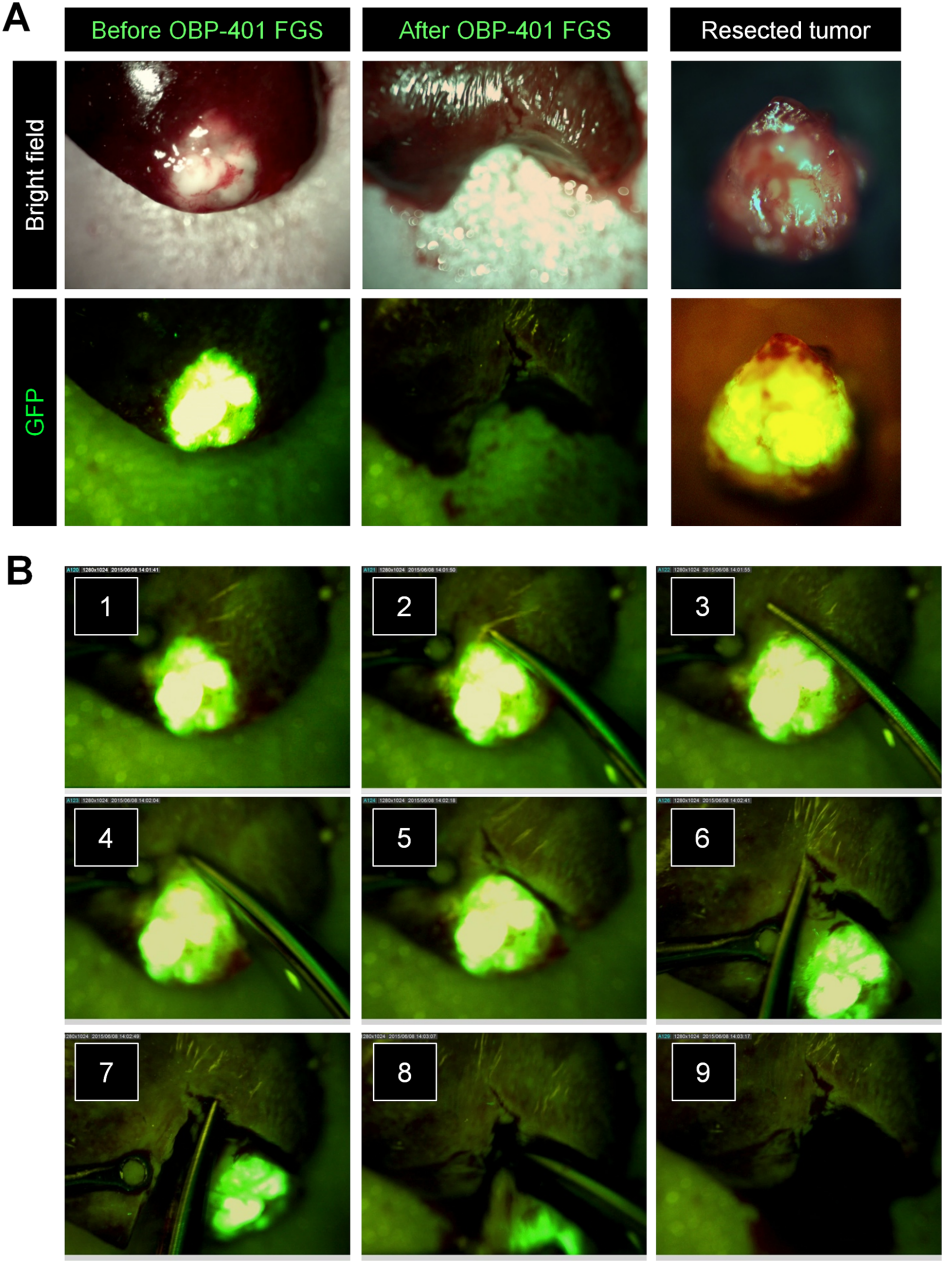
OBP-401-FGS of liver metastasis with the DinoLite hand-held fluorescence scope. **A.** Representative bright field and fluorescence images of solitary liver metastasis after labeling with OBP-401 (left). Representative bright field and fluorescence images of solitary liver metastasis with OBP-401-FGS (middle). Representative bright field and fluorescence images of resected metastasis labeled with OBP-401 (left). **B.** Procedure for OBP-401-FGS with the Dino-Lite hand-held fluorescence-scope.

GFP fluorescence of liver metastasis after OBP-401-GFP labeling was sufficiently bright to use the Dino-Lite hand-held fluorescence imager for FGS 3 days after OBP-401 infection (Fig 5A). OBP-401-GFP labeling made the tumor margin much clearer than under bright-light (Fig 5A). Using the Dino-Lite, the tumor margin was clearly visualized enabling complete resection of the solitary liver metastasis (Fig 5B, S1 Movie). Fluorescence imaging showed that there were no residual cancer cells after OBP-401-FGS with the Dino-Lite (Fig 5A).

### OBP-401-FGS Enables Complete Resection of the Liver Metastasis After Incomplete BLS Resection

**Fig 6:**
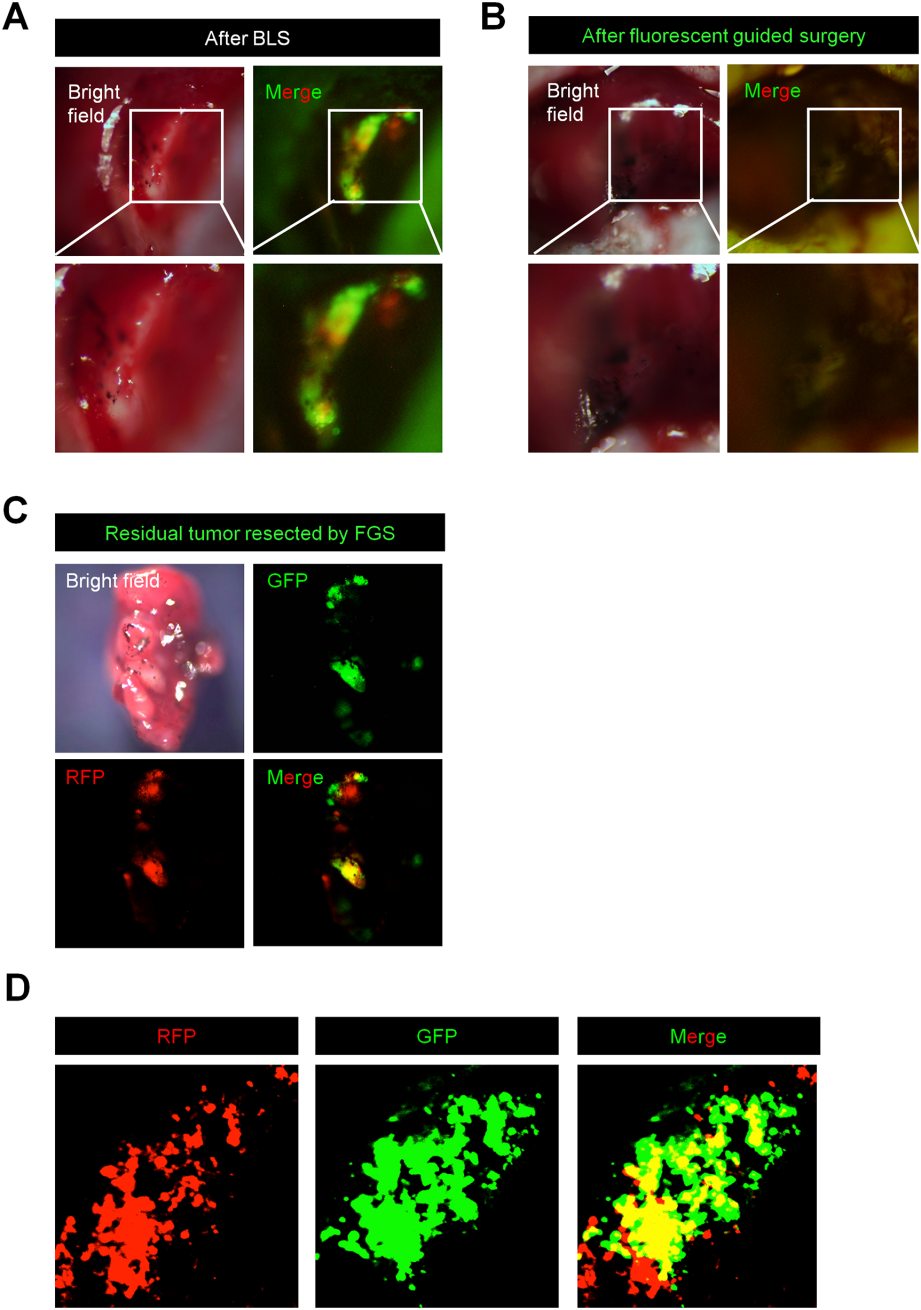
OBP-401 labeling visualizes residual cancer cells after BLS. **A.** Representative whole-liver images of non-infected orthotopic liver metastasis after bright-light surgery (BLS). **B.** Representative whole-liver images of orthotopic liver metastasis after OBP-401-FGS. **C.** Representative images of tumor resected by OBP-401-FGS. **D.** Representative single-cell image of residual tumor resected by OBP-401-FGS. **A-C**: Images were acquired with the OV100 whole body fluorescence imaging system. **D**: Images were acquired with the FV1000 confocal laser scanning microscope.

OBP-401 was injected into the liver metastasis 3 days before surgical resection. After BLS, both RFP and GFP fluorescence were detected in the surgical bed (Fig 6A). OBP-401 enabled detection of the residual cancer cells at the single cell level using fluorescence imaging with the FV1000 confocal microscope (Fig 6A). After OBP-401-FGS, there were no residual cancer cells (Fig 6B). Single-cell imaging, with the FV1000, demonstrated that GFP expression from OBP-401 co-localized with RFP expression of the cancer cells (Fig 6C and 6D).

### OBP-401-FGS Detects Satellite Liver Metastasis and Results in Complete Resection

**Fig 7:**
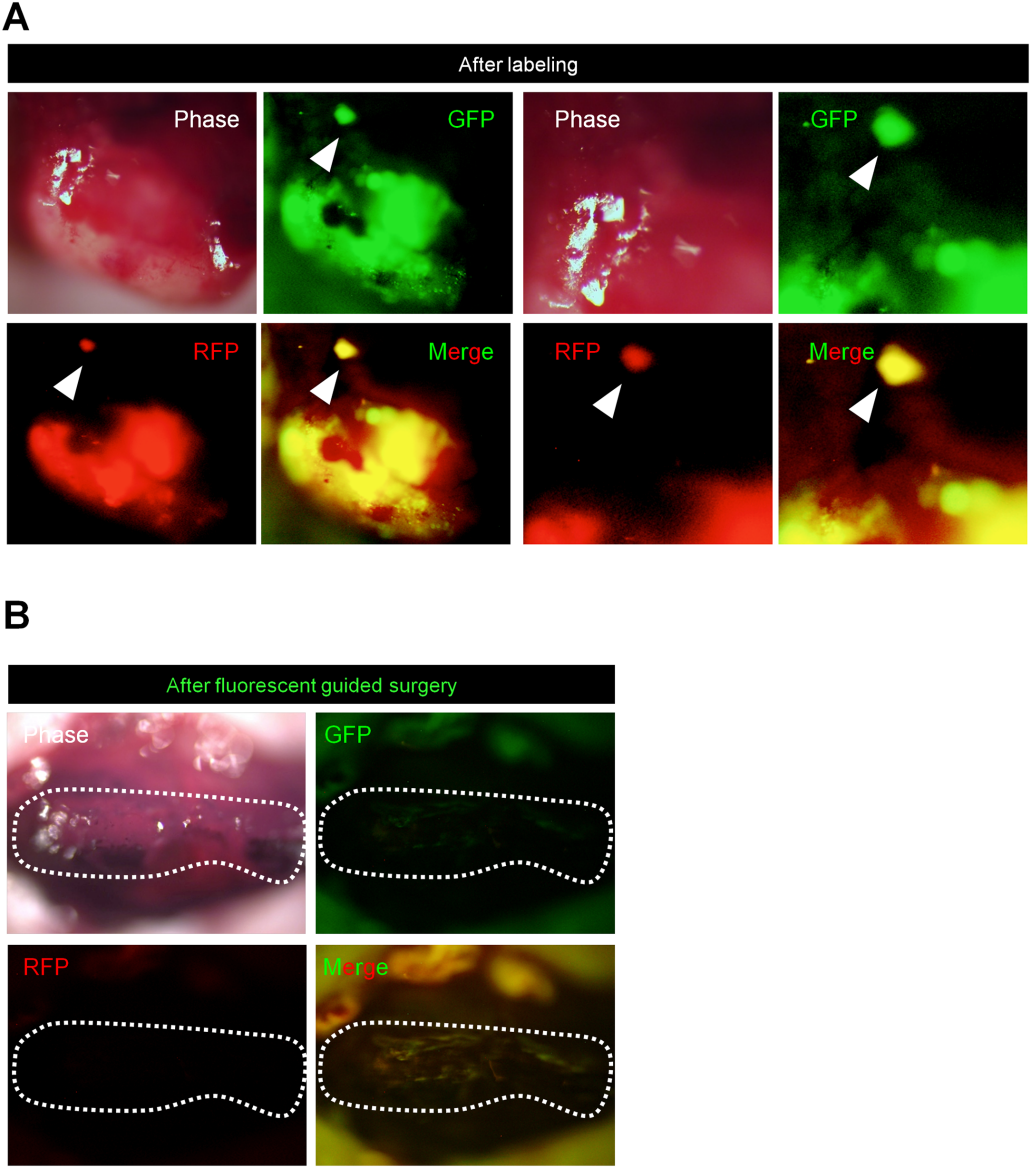
OBP-401 labeling visualizes satellite metastasis. OBP-401-labeled liver and satellite metastases were resected using BLS, and then residual cancer cells were resected using FGS. **A.** Representative low- (left) and high- (right) magnification images of the margin of the liver metastasis after OBP-401 labeling. **B.** Representative high-magnification images of complete resection beyond the margin after OBP-401-FGS.

OBP-401 was injected into the liver metastasis 3 days before surgical resection. OBP-401 GFP labeling enabled detection of a satellite as well as the main metastatic tumor (Fig 7A). OBP-FGS enabled complete resection of both the main and satellite metastasis (Fig 7B).

### Recurrence and Survival After OBP-401-FGS of the Solitary Liver Metastasis

**Fig 8:**
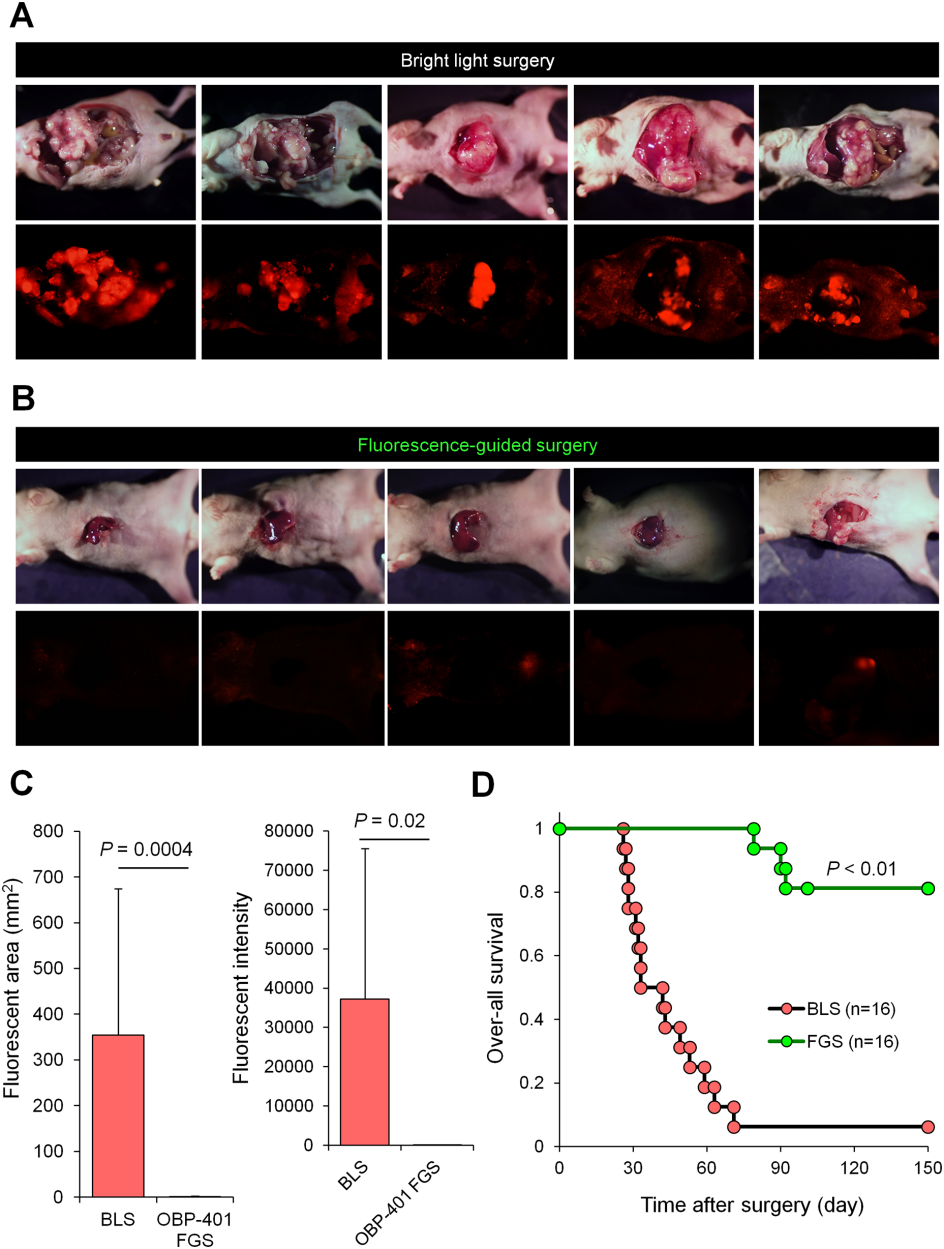
OBP-401-based FGS prolonged over-all survival compared with BLS. **A.** Representative images at necropsy of mice treated with BLS. **B.** Representative images 120 days after OBP-401-FGS. **C.** Comparison of fluorescent area of locally-recurrent tumors after BLS or OBP-401-FGS (left). Comparison of fluorescence intensity of locally-recurrent tumors after BLS or OBP-401-FGS (right). Fluorescence intensity is calculated with ImageJ software. Data are shown as average ± SD. N = 16. **D.** Kaplan-Meyer shows over-all survival after BLS or OBP-401-FGS.

Fifteen of sixteen mice that underwent BLS for liver metastasis had a large local recurrence (Fig 8A and 8C) (Table 1). Thirteen of sixteen mice which received OBP-401 did not have any recurrence (Fig 8B and 8C) (Table 1). Moreover, OBP-401-FGS significantly prolonged the over-all survival rate compared with BLS (Fig 8D).

**Table 1:** Numbers of animals with and without metastatic recurrence.

	Recurrence (positive)	Recurrence (negative)
BLS	15	1
FGS	3	13

P < 0.001

Pearson chi-square analysis was used to compare the rate of recurrence between BLS and OBP-401-FGS. Please see Materials and Methods for details.

Currently, single liver metastases are resected at some medical centers, but with frequent metastatic recurrence [17]. A greater extent of surgical resection can also lead to more rapid metastatic recurrence [18]. Therefore, precise resection of liver metastasis is necessary without extra wide margins in order to reduce recurrence.

The results of the present study demonstrate the power of OBP-401 to specifically label a liver metastasis in situ, enabling complete and precise resection by FGS, recurrence of 19% compared to 94% with BLS, and increased survival compared to BLS. The results of the present study suggest clinical promise for OBP-401 to improve outcome of liver metastasis, the often lethal aspect of colon and other cancers.

The parent virus of OBP-401, OBP-301, has proven safe in clinical trials [19]. Although OBP-301 can inhibit cancer growth in vivo, it does not eradicate the cancer cells [20, 21], as does the combination of OBP-401 and FGS. Clinical trials of the safety of OBP-401 are now called for, and if proven safe, OBP-401 FGS of liver cancer should proceed in the clinic.

## Supporting Information

S1 Checklist (PDF)

S1 Movie OBP-401 based-FGS of liver metastasis. Procedure for OBP-401-FGS with the Dino-Lite hand-held fluorescence-scope.
(MOV)

## Abbreviations

GFP: green fluorescent protein
RFP: red fluorescent protein
FGS: fluorescence-guided surgery
BLS: bright light surgery
